# Inactivation properties of sodium channel Na_v_1.8 maintain action potential amplitude in small DRG neurons in the context of depolarization

**DOI:** 10.1186/1744-8069-3-12

**Published:** 2007-05-31

**Authors:** T Patrick Harty, Stephen G Waxman

**Affiliations:** 1 Department of Neurology and Center for Neuroscience and Regeneration Research, Yale University School of Medicine, New Haven, CT 06510, USA, Rehabilitation Research Center, Veterans Affairs Connecticut Healthcare System, West Haven, CT 06516, USA

## Abstract

**Background:**

Small neurons of the dorsal root ganglion (DRG) express five of the nine known voltage-gated sodium channels. Each channel has unique biophysical characteristics which determine how it contributes to the generation of action potentials (AP). To better understand how AP amplitude is maintained in nociceptive DRG neurons and their centrally projecting axons, which are subjected to depolarization within the dorsal horn, we investigated the dependence of AP amplitude on membrane potential, and how that dependence is altered by the presence or absence of sodium channel Na_v_1.8.

**Results:**

In small neurons cultured from wild type (WT) adult mouse DRG, AP amplitude decreases as the membrane potential is depolarized from -90 mV to -30 mV. The decrease in amplitude is best fit by two Boltzmann equations, having V_1/2 _values of -73 and -37 mV. These values are similar to the V_1/2 _values for steady-state fast inactivation of tetrodotoxin-sensitive (TTX-s) sodium channels, and the tetrodotoxin-resistant (TTX-r) Na_v_1.8 sodium channel, respectively. Addition of TTX eliminates the more hyperpolarized V_1/2 _component and leads to increasing AP amplitude for holding potentials of -90 to -60 mV. This increase is substantially reduced by the addition of potassium channel blockers. In neurons from Na_v_1.8(-/-) mice, the voltage-dependent decrease in AP amplitude is characterized by a single Boltzmann equation with a V_1/2 _value of -55 mV, suggesting a shift in the steady-state fast inactivation properties of TTX-s sodium channels. Transfection of Na_v_1.8(-/-) DRG neurons with DNA encoding Na_v_1.8 results in a membrane potential-dependent decrease in AP amplitude that recapitulates WT properties.

**Conclusion:**

We conclude that the presence of Na_v_1.8 allows AP amplitude to be maintained in DRG neurons and their centrally projecting axons even when depolarized within the dorsal horn.

## Background

Peripheral nociceptors have a highly specialized function: transducing noxious stimuli into neural activity and transmitting that activity to the central nervous system. Encoded nociceptive information is transmitted via small diameter axons that originate from a population of small cell bodies (20–30 μm) contained within dorsal root ganglia (DRG), which send their central projections to the spinal cord dorsal horn. It is well-established that extracellular potassium levels ([K^+^]_o_) within the dorsal horn can rise significantly as a result of neuronal activity induced by many stimuli including peripheral injury and noxious stimuli [1]; these changes in [K^+^]_o _can, in turn, lead to depolarization which can inactivate sodium channels, producing conduction block of neurons and neuronal processes such as axons and their terminals [2-4]. Yet some forms of peripherally induced pain have a persistent quality, suggesting that the nociceptive afferent barrage can be maintained even in the face of this depolarization. This raises the possibility that the membranes of nociceptive DRG neurons and their centrally projecting axons are constructed so as to permit the conduction of action potentials even when depolarized.

Small DRG neurons, which include nociceptors, express five of the nine functional voltage-gated sodium channels that have been sequenced thus far, including three that are selectively expressed within DRG neurons. Three tetrodotoxin (TTX)-sensitive (TTX-s) channels, Na_v_1.1, 1.6 and 1.7 are expressed in adult small DRG neurons [5], as are the two TTX-resistant (TTX-r) channels, Na_v_1.8 and 1.9 [6-8]. These channels differ with regard to their biophysical properties. TTX-s channels in DRG neurons are characterized by relatively rapid activation and inactivation kinetics [9-13]. By comparison, the TTX-r channel Na_v_1.8 activates and inactivates more slowly [6,7]. Na_v_1.9 activates too slowly to contribute significantly to the upstroke of the action potential (AP), and its inactivation is so slow near activation threshold that the current is often referred to as persistent [14]. With regard to the voltage-dependence of channel opening and closing, TTX-r Na_v_1.8 channels in small DRG neurons have activation and steady-state fast inactivation functions that are depolarized compared to those of the TTX-s channels [6,7,9-13]. In DRG neurons, Na_v_1.7 channels have a relatively slow recovery from fast inactivation that presumably reduces its contribution to high frequency firing [15]. On the other hand, Na_v_1.7 channels have a relatively slow rate of inactivation from the closed state, which allows them to produce inward current in response to slow depolarizations.

Before the identification of the TTX-r channels Na_v_1.8 and Na_v_1.9, it was known that DRG neurons were different from other neurons because they could generate sodium-dependent action potentials (AP) in the presence of TTX [16-20]. Investigation of AP electrogenesis in small DRG neurons from Na_v_1.8 (-/-) animals by Renganathan et al [20] demonstrated that cells with a resting membrane potential (RMP) close to -50 mV produced small, graded responses to depolarization, whereas cells with a more hyperpolarized RMP (-65 mV) produced nearly full-sized APs. These findings suggested that TTX-s sodium channels were capable of generating AP's in small DRG neurons, but that most of these channels are inactivated at the typical RMP for these cells while, since the inactivation of Na_v_1.8 channels is more depolarized, more Na_v_1.8 channels are available at RMP to contribute to the AP. Similar conclusions based on voltage and current clamp recordings have been reached by other groups [21,22]. Na_v_1.8 is known to be expressed within the spinal cord dorsal horn, where it is present within the central terminals of primary sensory neurons [23,24]. In the present study we investigated the contribution of Na_v_1.8 to AP electrogenesis in DRG neurons from a different perspective, that of the reduction of AP amplitude in current clamp mode. We hypothesized that i) AP amplitude should decrease as a function of membrane potential, because membrane potential determines the degree of inactivation for the various sodium channel subtypes, and that ii)the presence of Na_v_1.8 should shift the voltage-dependence of AP amplitude in a depolarized direction, permitting relatively large APs to be generated even in depolarized neurons.

## Results

**Figure 1:**
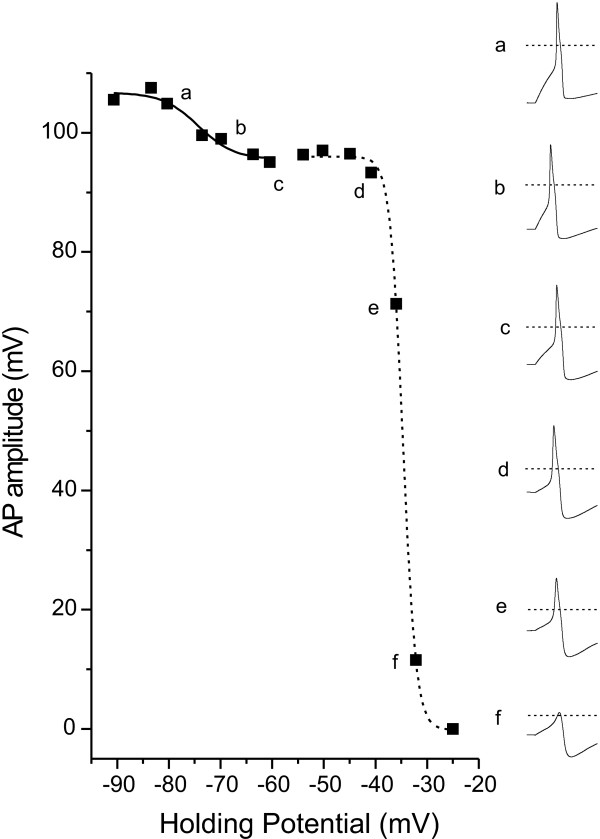
The voltage dependent decrease in AP amplitude recorded from a small diameter, WT DRG neuron is best fit by 2 Boltzmann equations. APs were elicited by threshold stimulation while holding the cell membrane potential between -90 and -25 mV. Inset waveforms on the right represent responses elicited at the membrane holding potential indicated by the small letters (a-f). The dotted lines in the insets indicate 0 mV. The solid and dashed lines in the main figure represent Boltzmann equation fits with V_1/2 _values of -74.3 mV and -34.7 mV, respectively. For this cell, the contribution of the more hyperpolarized component accounted for 10% of the decrease in AP amplitude.

We first tested the dependence of AP amplitude on the membrane potential of small (< 25 um) DRG neurons from adult wild type mice. This was achieved in current clamp mode, by using injected current to hold the cell at a series of membrane potentials starting at -90 mV, with successive depolarizing steps of approximately 5 mV. At each holding potential (V_h_), the cell was stimulated with a positive-going square pulse of current (100 ms) starting just below threshold and proceeding in 10 pA intervals to a level 40–50 pA beyond threshold. A plot of AP amplitude (see Methods section for details on measurement procedure) and V_h _(Figure 1) revealed that AP amplitude decreased gradually as a function of cell membrane potential, but with a clear shoulder in the relationship. The voltage-dependent decrease is clearly visible in representative AP responses (waveform insets a-e in Figure 1). The relationship between AP amplitude and V_h _was best fit with 2 Boltzmann equations, one having a mean V_1/2 _of -73 ± 2 mV and accounting for 27 ± 6% of the decrease in AP amplitude, the other having a mean V_1/2 _of -37 ± 2 mV and accounting for 81 ± 6 % of the decrease in AP amplitude. The more hyperpolarized V_1/2 _is consistent with the voltage-dependence of steady-state fast inactivation that is characteristic of the TTX-s sodium channels present in small DRG neurons (V_1/2_'s of -64 to -75 mV), while the more depolarized V_1/2 _is consistent with a contribution from TTX-r channels, which have fast inactivation V_1/2_'s of -30 to -45 mV [6,7,9-13].

**Figure 2:**
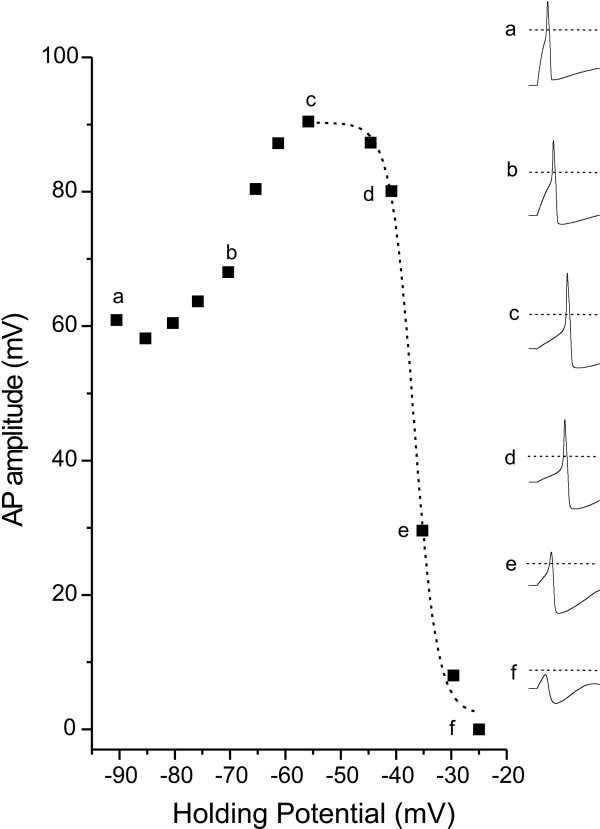
Block of TTX-s sodium channels alters the voltage dependent decrease in AP amplitude. Responses from a single small DRG neuron in the presence of 300 nM TTX (inset waveforms a-f on the right) demonstrate that AP amplitude increases with depolarization of membrane holding potential from -90 to -60 mV (inset waveforms a-c). From holding potentials of -60 to -25, AP amplitude decreases with depolarization (inset waveforms c-f), and this decrease can be fit with a single Boltzmann equation (dashed line in the main figure, V_1/2 _= -36.8 mV). The dotted lines in inset waveforms indicate 0 mV.

To investigate the hypothesized contribution of TTX-s currents to AP amplitude, we performed the same analysis of AP amplitude and V_h _in DRG from WT mice with the inclusion of TTX (300 nM). As seen in Figure 2, for V_h _values from approximately -50 mV to -25 mV, there was a gradual decrease in AP amplitude that was well fit with a single Boltzmann equation. The mean V_1/2 _characterizing this voltage-dependence was -36 ± 2 mV, which was similar to the depolarized component in absence of TTX and consistent with the elimination of TTX-s currents responsible for the decrease in AP amplitude at more hyperpolarized membrane potentials. An unexpected finding was that AP amplitude actually increased in amplitude as V_h _was increased from -90 to about -60 mV. The average AP amplitude at a V_h _of -90 mV was 46.2 ± 4.7 mV, representing a decrease of 45 ± 4% compared to the maximum AP amplitude. In the absence TTX-s sodium currents and any other influence on AP amplitude, it would be expected that AP amplitude would remain constant at these hyperpolarized potentials because TTX-r sodium channels would be in closed and activatable states.

**Figure 3:**
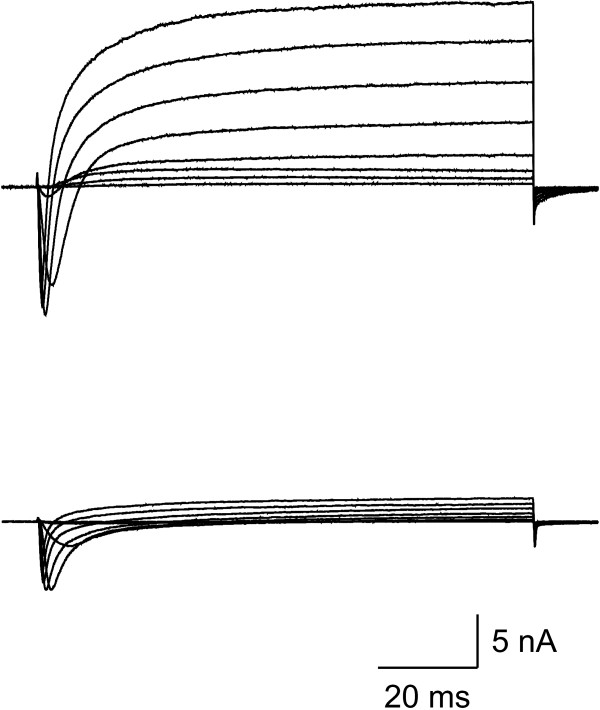
Potassium channel blockers reduce outward currents. Voltage clamped currents in response to depolarizing voltage steps from a holding potential of -100 mV are shown for two separate small DRG neurons. Cells were recorded in the absence (top) and presence (bottom) of the potassium channel blockers TEA and 4-AP.

**Figure 4:**
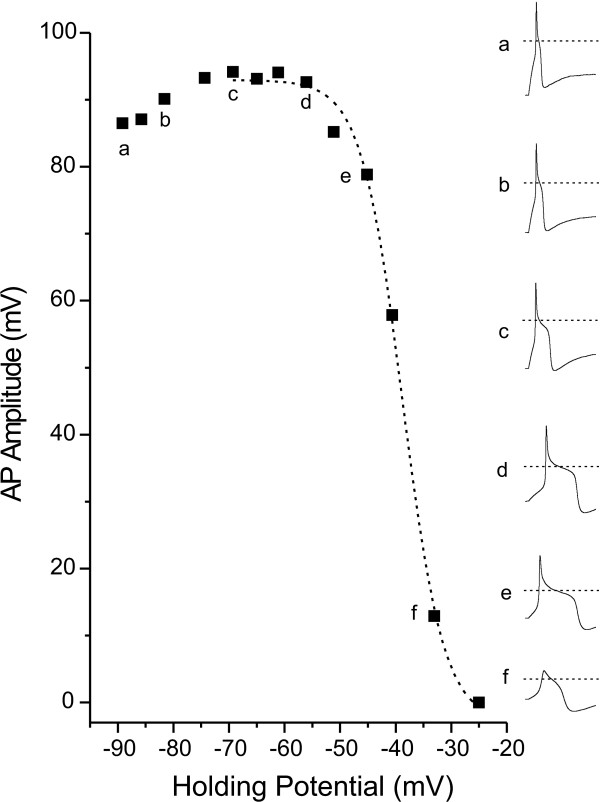
Potassium channel blockers reduce the increase in AP amplitude observed in the presence of TTX for hyperpolarized holding potentials. Responses from a single small DRG neuron (inset waveforms a-f on the right) demonstrate that the increase in AP amplitude observed in the presence of TTX with depolarization of membrane holding potential from -90 to -60 mV (inset waveforms a-c) is much smaller in the presence of TEA and 4-AP. In contrast, the decrease in AP amplitude with depolarization from holding potentials of -60 to -25 mV (inset waveforms c-f) is unchanged in the presence of TEA and 4-AP. The decrease can be fit with a single Boltzmann equation (dashed line in main figure, V_1/2 _= -38.8 mV). The dotted lines in inset waveforms indicate 0 mV.

We hypothesized that, in addition to voltage-gated sodium channels, a strong influence on AP amplitude would be from outward potassium currents. To test this hypothesis, we performed the same AP amplitude/V_h _analysis in the presence of TTX (300 nM), TEA (25 mM) and 4-AP (3 mM) to block a significant portion of outward potassium current. Just before switching to current clamp recording mode, the extent of potassium channel block was determined by holding cells at -100 mV and stepping for 100 ms to membrane voltages between -40 and +25. An example of currents produced by this protocol for two cells, one in the absence (A) and one in the presence (B) of TEA and 4-AP are shown in Figure 3. In the absence of TEA and 4-AP, maximum outward current often exceeded 20 nA, thus saturating the amplifier. To quantify the extent of block, maximum outward current was measured at steps to 0 mV. For cells in the absence of TEA and 4-AP, the maximum outward current at 0 mV was 13.8 ± 1.9 nA (N = 8), whereas for cells in the presence of TEA and 4-AP the maximum outward current was 1.4 ± 0.3 nA (N = 6), representing a reduction of 90%. For this group of cells tested in the presence of TEA, 4-AP as well as TTX, AP amplitude decreased gradually for V_h _values between -60 and -20 (Figure 4). This voltage dependence was fit with a single Boltzmann equation producing a V_1/2 _of -40 ± 1.3 mV, which was similar to the single component fit for WT neurons in the presence of TTX. In addition, the increase in AP amplitude observed in the presence of TTX alone for V_h _from -90 to -60 mV was significantly reduced. The average AP amplitude at V_h _-90 mV was 83.6 ± 6.4 mV, representing a decrease of 9 ± 3% of the maximum AP amplitude. Thus, blocking 90% of the outward potassium current led to a reduction in the decrease in AP amplitude at the most hyperpolarized membrane potential (-90 mV) from 45 to 9%. The remaining decrease may be attributable to the unblocked outward current or additional factors that can influence AP amplitude.

**Figure 5:**
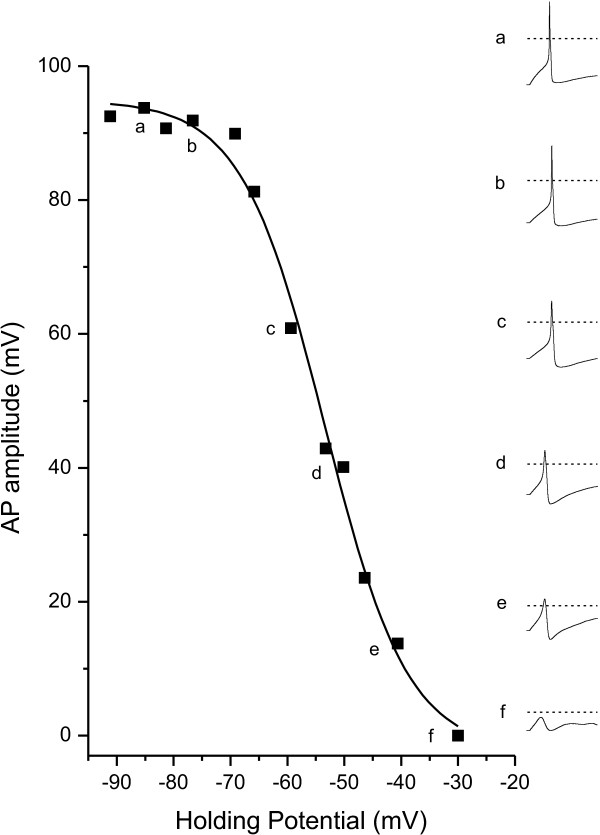
The absence of Na_v_1.8 sodium channels alters the voltage dependent decrease in AP amplitude. Responses from a small DRG neuron cultured from a Na_v_1.8(-/-) mouse are shown as inset waveforms on the right (a-f). In these neurons, the decrease in AP amplitude as membrane holding potential is depolarized from -90 to -30 mV is best fit by a single Boltzmann equation (solid line in main figure). The V_1/2 _(-53.4 mV for this cell) is about 20 mV more depolarized than the TTX-s component in small DRG neurons from WT mice. The dotted lines in inset waveforms indicate 0 mV.

**Figure 6:**
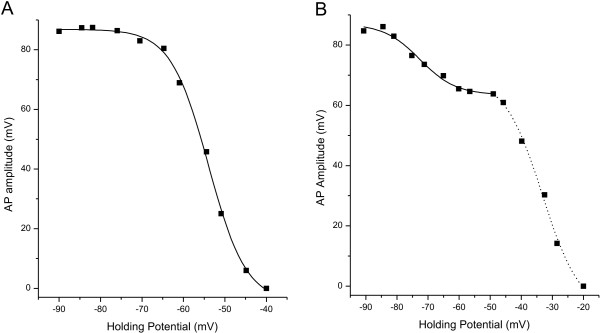
**Transfection with Na_v_1.8 produces two populations of   cells with respect to voltage-dependence of AP amplitude.**  Data are   from two small DRG neurons from Na_v_1.8(-/-) mice transfected with   Nav1.8.    The cell in (A) is representative of a population of cells   (N = 7) for which the decrease in AP amplitude was best fit with   single Boltzmann equation having a  V_1/2_  of -53.9 mV (solid line).     The cell in (B) is representative of a population of cells (N = 8) for   which the decrease in AP amplitude was best fit  by two Boltzmann   equations, with  V_1/2_  values of -72.6  mV (solid line) and -33.3 mV   (dashed line).

To further investigate the contribution of TTX-r currents to the decrease in AP amplitude associated with the more depolarized V_1/2_, we applied the AP amplitude/V_h _analysis to small DRG neurons obtained from Na_v_1.8(-/-) mice transfected with either GFP alone, or GFP plus a construct containing Na_v_1.8. As shown in Figure 5, AP amplitude in cells transfected with GFP alone decreased gradually as V_h _increased from -90 to -30 mV, and this decrease was best fit with a single Boltzmann equation. The mean V_1/2 _for 10 cells was -55.0 ± 1.5 mV, a value that is depolarized by about 20 mV compared to the TTX-s component in WT neurons. When Na_v_1.8 was transfected back into small DRG neurons from Na_v_1.8(-/-) animals, analysis of the decrease in AP amplitude as a function of V_h _two populations revealed two populations: for one group, decrease in AP amplitude was best fit by a single Boltzmann equation (Figure 6A) having a mean V_1/2 _of -52 ± 1.5 mV (N = 7). This value was not statistically different from the V_1/2 _value for the cells from Na_v_1.8(-/-) animals transfected with GFP alone. Presumably, this group of cells did not express the Na_v_1.8 construct. In a second group of cells (N = 8), the decrease in AP amplitude was best fit by two Boltzmann equations with mean V_1/2_'s of -71 ± 2.1 mV and -35.6 ± 3.0 mV. These values are not significantly different from the corresponding values for the small DRG neurons from WT animals. In addition, the portion of the decrease in AP amplitude accounted for by each V_1/2 _was similar to the WT data (24 ± 3% for the more hyperpolarized component, 77 ± 3% for the more depolarized component). These data indicate that the shift in V_1/2 _observed in the Na_v_1.8(-/-) neurons, presumably dependent on the fast inactivation of TTX-s sodium currents, is due to the absence of Na_v_1.8 channels.

## Discussion

This work was motivated by the question of how AP conduction can be maintained in the central projections of nociceptive DRG neurons which course through the dorsal horn where they are subjected to depolarization [1,2] and by the observations that Na_v_1.8 channels are present within the central terminals of primary sensory neurons within the dorsal horn [23,24] and that the amplitude of APs recorded from small DRG neurons is dependent on the holding potential of the cell, and that this voltage dependence is best fit with two Boltzmann equations. Because sodium channels provide the primary source of current for the AP upstroke, the latter finding suggested a method for investigating the contribution of the various sodium channels expressed by small DRG neurons to AP generation. The V_1/2 _values for the two Boltzmann fits (-73 mV and 37 mV)are similar to the V_1/2 _values for fast steady-state inactivation associated with TTX-s channels (-65 to -75 mV) and Na_v_1.8 TTX-r channels (-30 to -40 mV), respectively, that are expressed in small DRG neurons [6,7,9-13]. The substantial contribution of the component with the more depolarized V_1/2 _to AP amplitude (81%), is consistent with previous estimates of the relative contribution of Na_v_1.8 current to AP amplitude. Using the Goldman-Hodgkin-Katz equation to estimate sodium ion permeability during the AP in DRG from Na_v_1.8(+/+) and Na_v_1.8(-/-) animals, Renganathan concluded that Na_v_1.8 channels contribute 80–90% of the current that flows at the peak of the AP [20]. Blair and Bean isolated TTX-s, TTX-r and high voltage-activated calcium currents in small DRG neurons stimulated with a simulated AP voltage protocol [21]. They estimated that TTX-r currents contributed 60% of the AP upstroke, as compared to 40% for the TTX-s component.

To confirm the contribution of TTX-s channels, we determined the voltage-dependence of AP amplitude in the presence of 300 nM TTX. For V_h _values of approximately -60 to -20 mV, AP amplitude decreased gradually, and this voltage-dependence was well fit with a single Boltzmann equation having a V_1/2 _of -36 mV. The absence of the more hyperpolarized component demonstrates a contribution of TTX-s sodium currents. In place of a decrease in AP amplitude, we observed that AP amplitude increased for Vh values of -90 to -60 mV. It is unlikely that an increasing availability of TTX-r channels could account for increasing AP amplitude in this voltage range because it does not fall with the range for steady-state fast inactivation of these channels. Alternatively, we hypothesized that outward current generated by potassium channel opening might be responsible for the change in AP amplitude in the voltage range. To test this hypothesis, we recorded AP amplitude under conditions known to block the majority of voltage-gated potassium channels (TEA and 4-AP). Estimates of outward current amplitude obtained before switching to current clamp mode (see Methods) indicated that 80–90% of the outward current produced by depolarizing voltage steps was blocked. In current clamp mode, analysis of AP amplitude and V_h _revealed that the 45% increase in AP amplitude from -90 to -60 mV in the presence of TTX alone decreased to only 9% in the presence of TEA and 4-AP, representing a reduction of 80%. Although we cannot state with certainty the identity of the potassium channels reducing AP amplitude from -90 to -60 mV, possible candidates are the inwardly rectifying current I_IR _[25] and I_h _[26], a slowly activated inward current, both of which are initiated by membrane hyperpolarization. It is interesting to consider the possibility that one of the roles of TTX-s sodium currents in small DRG neurons is to boost AP amplitude at these hyperpolarized membrane potentials.

In the absence of well-established blockers of the Na_v_1.8 sodium current, we used DRG neurons from Na_v_1.8(-/-) animals to further investigate the identity of the currents contributing to the depolarized V_1/2 _characterizing AP amplitude reduction. Previous studies have demonstrated that small DRG neurons from these animals completely lack the slow TTX-r current produced by Na_v_1.8 channels [27]. AP amplitude in these cells decreased with a voltage dependence that was well fit by a single Boltzmann equation with a V_1/2 _of -55 mV. This value represents a depolarizing shift of 20 mV compared to the V_1/2 _for the TTX-s component in WT neurons. One possible explanation is that the inactivation properties of the remaining TTX-r sodium channels, Na_v_1.9, are combining with those of TTX-s sodium channels to produce a single depolarized V_1/2_. The midpoint of steady-state fast inactivation for Na_v_1.9 in DRG cells ranges from -44 to -54 mV [14,28]. However, the slow onset of Na_v_1.9 channel openings indicates that these channels make only a minor contribution to AP amplitude [22,28]. Akopian et al [27] observed an up-regulation of Na_v_1.7 in Na_v_1.8(-/-) DRG neurons; however, this would not account for the shift in V_1/2 _because the V_1/2 _for steady-state fast inactivation of Na_v_1.7, -71 mV to -78 mV [15,29], is similar to the more hyperpolarized V_1/2 _associated with the TTX-s component of AP decrease in WT neurons. An alternative explanation for the shift in the V_1/2 _of the TTX-s component is that the inactivation properties of TTX-s sodium channels are modified in DRG neurons of Na_v_1.8(-/-) animals. Such a modification has been observed in a previous study [20]. In a comparison of TTX-s currents in small DRG neurons from Na_v_1.8(+/+) and Na_v_1.8(-/-) mice, the authors observed a 20 mV depolarizing shift in the voltage dependence of fast inactivation. The mechanism for this shift in TTX-s voltage dependence of inactivation was not determined. Possible mechanisms include G-protein activation, which has been shown to depolarize inactivation V_1/2 _of Na_v_1.8 currents by 3–4 mV in DRG neurons [30], the presence of arachidonic acid, which hyperpolarizes the inactivation V_1/2 _of both TTX-s and TTX-r currents in DRG neurons [31] and tyrosine kinase phosphorylation, which has been shown to both depolarize the V_1/2 _of fast inactivation in cardiac Na_v_1.5 channels in HEK293 cells, [32] and hyperpolarize the V_1/2 _of fast inactivation in sodium currents of differentiated PC-12 cells [33]. Because expression of β subunits can influence sodium channel inactivation properties [34], differential expression of β subunits between Na_v_1.8(+/+) and Na_v_1.8(-/-) animals might also account for the observed shift. Irrespective of the modulatory process involved, our additional results with transfection of Na_v_1.8 into cells from Na_v_1.8(-/-) animals demonstrates that the process is not permanent and is dependent on the absence of Na_v_1.8 channels. After transfections, AP amplitude dependence on V_h _was again fit best by two Boltzmann equations in half of the cells, and the V_1/2 _values (-71 and -36 mV) were similar to values for the wild type DRG neurons. These results indicate that the reintroduced Na_v_1.8 channels led to a shift in the inactivation voltage-dependence of the TTX-s component back to the WT value.

## Conclusion

In summary, we have shown that the AP amplitude of small DRG neurons decreases as a function of membrane depolarization, and that this relationship is best fit with two Boltzmann components. The V_1/2 _characterizing the more hyperpolarized component is similar to the V_1/2 _associated with TTX-s sodium channel steady-state fast inactivation and is eliminated in the presence of TTX. The V_1/2 _characterizing the more depolarized component is similar to the V_1/2 _of steady-state fast inactivation for Na_v_1.8. In small DRG neurons from Na_v_1.8(-/-) animals, the voltage-dependence of the AP amplitude decrease is characterized by a single Boltzmann component with a V_1/2 _of -55 mV, a value that is depolarized by about 20 mV compared to the TTX-s component in neurons from WT animals. We believe that this shift is due to a shift in the inactivation properties of TTX-s sodium channels in small DRG neurons from Na_v_1.8(-/-) animals, but that this shift is not permanent. When these same cells are transfected with Na_v_1.8, the voltage-dependence of AP amplitude recapitulates the voltage-dependence observed in neurons from WT animals. Taken together, these results suggest that the expression of Na_v_1.8 within nociceptive DRG neurons permits the central projections of these cells to conduct APs even when depolarized within the dorsal horn.

## Methods

### Animal care

All animal care and surgical procedures followed a protocol approved by the Animal Care and Use Committee of Yale University. A colony of Na_v_1.8(-/-) mice was raised from a breeding pair of Na_v_1.8(+/-) mice [27] generously provided by John Wood (University College, London). Animals were housed in a pathogen-free area at the Veterans Affairs Medical Center in West Haven, CT and fed ad libitum.

### Culturing and transfection of DRG neurons

DRGs were harvested from deeply anesthetized (ketamine/xylazine; 80/10 mg/kg; i.p.) adult (4–6 weeks) C57BL6 mice or adult Na_v_1.8(-/-) mice (in C57/BL6 genetic background). Neurons were isolated as previously described [35]. Briefly, tissue was enzymatically digested in collagenase A (1 mg/ml, Roche) and sterile complete saline solution (CSS) for 20 min at 37°C, and then for 20 min in CSS solution with collagenase D (1 mg/ml, Roche). The pRK-Na_v_1.8 plasmid used for transfections of Na_v_1.8 into DRG from Na_v_1.8(-/-) mice was a gift from John Wood (University College, London). The green fluorescent protein plasmid pEGFP was purchased from Clontech (Palo Alto, CA). Sodium channel and GFP constructs (channel:GFP ratio of 5:1) were electroporated into DRG neurons using Nucleofector Solution (Amaxa, Gaithersburg, MD) as previously described [36,37]. Transfected DRG neurons were incubated at 37°C in Ca^2+^- and Mg^2+^-free culture medium (DMEM plus 10% fetal calf serum) for 5 min. to increase cell viability. The cell suspension was then diluted in culture medium, plated on 12 mm circular coverslips coated with laminin and poly-ornithine, and incubated at 37°C and 5% CO_2_.

### Current and voltage clamp electrophysiology

Small (20–25 μm) DRG neurons were used for current clamp recording after 24–40 hours in culture (with or without transfection). Electrodes had a resistance of 1–2 MΩ when filled with the pipette solution, which contained (in mM): 140 KCl, 0.5 EGTA, 5 HEPES and 3 Mg-ATP (pH 7.3 with KOH, adjusted to 320 mOsm with dextrose). The extracellular solution contained (in mM): 140 NaCl, 3 KCl, 2 MgCl_2_, 2 CaCl_2_, 10 HEPES (pH 7.3 with NaOH, adjusted to 320 mOsm with dextrose). The whole-cell recording configuration was obtained in voltage clamp mode with an Axopatch 200B amplifier (Molecular Devices, Sunnyvale, CA). Data was stored on a personal computer via a Digidata 1322a A/D converter (Molecular Devices) at an acquisition rate of 50 kHz with a lowpass Bessel filter setting of 5 kHz. In voltage clamp recording mode, voltage errors were minimized with 85% series resistance compensation, and linear leak currents and capacitance artifacts were subtracted out using the P/N method provided by Clampex (Molecular Devices) acquisition software. Clampfit (Molecular Devices) and Origin (Microcal Software, Northampton, MA) were used for data analysis. To obtain an estimate of inward and outward current amplitudes, two activation protocols were applied before proceeding to the current clamp recording mode. Both protocols involved steps to a range of membrane potentials from -45 to 40 mV, in 5 mV increments. For the first protocol, cells were held at -50 mV to eliminate the majority of TTX-s sodium channels. For the second protocol, cells were held at -100 mV to activate both TTX-s and TTX-r channels. A rough estimate of TTX-s currents was obtained by subtraction of the currents obtained from the two protocols. After switching to current clamp recording mode, passive and active cell properties were determined (RMP, input resistance, spike amplitude and threshold at RMP). Cells with RMPs more negative than -35 mV and overshooting action potentials (>85 mV RMP to peak) were used for further data collection. Stimulation threshold at RMP was determined by a applying a series of 100 ms depolarizing current steps starting below threshold and increasing in 5 pA increments. Threshold was defined as the smallest current amplitude that produced an AP. To investigate the voltage dependence of AP amplitude, current injection was used to hold cells at membrane potentials (V_h_) starting at -90 mV and depolarizing by 10 mV until an overshooting AP could not be elicited. At each V_h_, threshold was determined and AP amplitude for the response at threshold + 10 pA was used for the analysis. AP amplitude was defined as the difference between the peak and the onset of the AP. Onset of the AP was further defined as the point at which dV/dt for the AP was greater than 5 mV/ms (this value was chosen to be approximately 2 times the maximum baseline noise of the dV/dt trace). AP amplitude was plotted as a function of V_h _and fit with either 1 or 2 Boltzmann equations of the form V_AP_/V_APmax _= 1/(1 + exp((V_h _- V_1/2_)/k)), for which V_AP _is the AP amplitude, V_APmax _is the maximum AP amplitude, V_h _is the holding potential, V_1/2 _is the V_h _at which the AP amplitude is 50% of maximum, and k is the slope of the fit. Goodness of fit for 1 and 2 Boltzmann equations was determined by eye. Data is expressed as mean ± sem, and for all data comparisons, Student's t-test was used to assess statistical differences between means from different groups.

## Competing interests

The author(s) declare that they have no competing interests.

## Authors' contributions

SGW and TPH planned the experiments. TPH conducted all the experiments. Both authors contributed to the writing of the paper.

## References

[B1] Svoboda J, Motin V, Hájek I, Syková E (1988). Increase in extracellular potassium level in rat spinal dorsal horn induced by noxious stimulation and peripheral injury. Brain Res.

[B2] Vyklický L, Syková E, Kříž N, Ujec E (1972). Post-stimulation changes of extracellular potassium concenteration in the spinal cord of the rat. Brain Res.

[B3] Wall PD (1994). Control of impulse conduction in long range branches of afferents by increases and decreases of primary afferent depolarization in the rat. Eur J Neurosci.

[B4] Malenka RC, Kocsis JD, Ransom BR, Waxman SG (1981). Modulation of parallel fiber excitability by postsynaptically mediated changes in extracellular potassium. Science.

[B5] Black JA, Dib-Hajj S, McNabola K, Jeste S, Rizzo MA, Kocsis JD, Waxman SG (1996). Spinal sensory neurons express multiple sodium channel alpha-subunit mRNAs. Mol Brain Res.

[B6] Akopian AN, Sivilotti L, Wood JN (1996). A tetrodotoxin-resistant voltage-gated sodium channel expressed by sensory neurons. Nature.

[B7] Sangameswaran L, Delgado SG, Fish LM, Koch BD, Jakeman LB, Stewart GR, Sze P, Hunter JC, Eglen RM, Herman RC (1996). Structure and function of a novel voltage-gated, tetrodotoxin-resistant sodium channel specific to sensory neurons. J Biol Chem.

[B8] Dib-Hajj S, Black JA, Cummins TR, Waxman SG (2002). NaN/Nav1.9: a sodium channel with unique properties. Trends Neurosci.

[B9] Caffrey JM, Eng DL, Black JA, Waxman SG, Kocsis JD (1992). Three types of sodium channels in adult rat dorsal root ganglion neurons. Brain Res.

[B10] Elliott AA, Elliott JR (1993). Characterization of TTX-sensitive and TTX-resistant sodium currents in small cells from adult rat dorsal root ganglia. J Physiol.

[B11] Kostyuk PG, Veselovsky NS, Tsyndrenko AY (1981). Ionic currents in the somatic membrane of rat dorsal root ganglion neurons-I. Sodium currents. Neuroscience.

[B12] Roy ML, Narahashi T (1992). Differential properties of tetrodotoxin-sensitive and tetrodotoxin-resistant sodium channels in rat dorsal root ganglion neurons. J Neurosci.

[B13] Ogata N, Tatebayashi H (1993). Kinetic analysis of two types of Na+ channels in rat dorsal root ganglia. J Physiol.

[B14] Cummins TR, Dib-Hajj SD, Black JA, Akopian AN, Wood JN, Waxman SG (1999). A novel persistent tetrodotoxin-resistant sodium current in SNS-null and wild-type small primary sensory neurons. J Neurosci.

[B15] Cummins TR, Howe JR, Waxman SG (1998). Slow closed-state inactivation: a novel mechanism underlying ramp currents in cells expressing the hNE/PN1 sodium channel. J Neurosci.

[B16] Jeftinija S (1994). The role of tetrodotoxin-resistant sodium channels of small primary afferent fibers. Brain Res.

[B17] Gaumann DM, Brunet PC, Jirounek P (1992). Clonidine enhances the effects of lidocaine on C-fiber action potential. Anesth Analg.

[B18] Kobayashi J, Ohta M, Terada Y (1993). C fiber generates a slow Na+ spike in the frog sciatic nerve. Neurosci Lett.

[B19] Leffler A, Herzog RI, Dib-Hajj SD, Waxman SG, Cummins TR (2005). Pharmacological properties of neuronal TTX-resistant sodium channels and the role of a critical serine pore residue. Pflugers Arch.

[B20] Renganathan M, Cummins TR, Waxman SG (2001). Contribution of Na[v]1.8 sodium channels to action potential electrogenesis in DRG neurons. J Neurophysiol.

[B21] Blair NT, Bean BP (2002). Roles of tetrodotoxin [TTX]-sensitive Na+ current, TTX-resistant Na+ current, and Ca2+ current in the action potentials of nociceptive sensory neurons. J Neurosci.

[B22] Matsutomi T, Nakamoto C, Zheng T, Kakimura J, Ogata N (2006). Multiple types of Na[+] currents mediate action potential electrogenesis in small neurons of mouse dorsal root ganglia. Pflugers Arch.

[B23] Novakovic SD, Tzoumaka E, McGivern JG, Haraguchi M, Sangameswaran L, Gogas KR, Eglen RM, Hunter JC (1998). Distribution of the tetrodoxin-resistant sodium channel PN3 in rat sensory neurons in normal and neuropathic conditions. J Neurosci.

[B24] Amaya F, Decosterd I, Samad TA, Plumpton C, Tate S, Mannion RJ, Costigan M, Woolf CJ (2000). Diversity of expression of the sensory neuron-specific TTX-resistant voltage-gated sodium ion channels SNS and SNS2. Molec and Cell Neurosci.

[B25] Scroggs RS, Todorovic SM, Anderson EG, Fox AP (1994). Variation in IH, IIR, and ILEAK between acutely isolated adult rat dorsal root ganglion neurons of different size. J Neurophysiol.

[B26] Tu H, Deng L, Sun Q, Yao L, Han JS, Wan Y (2004). Hyperpolarization-activated, cyclic nucleotide-gated cation channels: roles in the differential electrophysiological properties of rat primary afferent neurons. J Neurosci Res.

[B27] Akopian AN, Souslova V, England S, Okuse K, Ogata N, Ure J, Smith A, Kerr BJ, McMahon SB, Boyce S, Hill R, Stanfa LC, Dickenson AH, Wood JN (1999). The tetrodotoxin-resistant sodium channel SNS has a specialized function in pain pathways. Nat Neurosci.

[B28] Herzog RI, Cummins TR, Waxman SG (2001). Persistent TTX-resistant Na+ current affects resting potential and response to depolarization in simulated spinal sensory neurons. J Neurophysiol.

[B29] Herzog RI, Cummins TR, Ghassemi F, Dib-Hajj SD, Waxman SG (2003). Distinct repriming and closed-state inactivation kinetics of Nav1.6 and Nav1.7 sodium channels in mouse spinal sensory neurons. J Physiol.

[B30] Saab CY, Cummins TR, Waxman SG (2003). GTP gamma S increases Nav1.8 current in small-diameter dorsal root ganglia neurons. Exp Brain Res.

[B31] Lee GY, Shin YK, Lee CS, Song JH (2002). Effects of arachidonic acid on sodium currents in rat dorsal root ganglion neurons. Brain Res.

[B32] Ahern CA, Zhang JF, Wookalis MJ, Horn R (2005). Modulation of the cardiac sodium channel Nav1.5 by Fyn, a Src family tyrosine kinase. Circ Res.

[B33] Hilborn MD, Vaillancourt RR, Rane SG (1998). Growth factor receptor tyrosine kinases acutely regulate neuronal sodium channels through the src signaling pathway. J Neurosci.

[B34] Vijayaragavan R, Powell AJ, Kinghorn IJ, Chahine M (2004). Role of auxiliary beta1-, beta2-, and beta3-subunits and their interaction with Na[v]1.8 voltage-gated sodium channel. Biochem Biophys Res Commun.

[B35] Rizzo MA, Kocsis JD, Waxman SG (1994). Slow sodium conductances of dorsal root ganglion neurons: intraneuronal homogeneity and interneuronal heterogeneity. J Neurophysiol.

[B36] Dib-Hajj SD, Rush AM, Cummins TR, Hisama FM, Novella S, Tyrrell L, Marshall L, Waxman SG (2005). Gain-of-function mutation in Nav1.7 in familial erythromelalgia induces bursting of sensory neurons. Brain.

[B37] Rush AM, Dib-Hajj SD, Liu S, Cummins TR, Black JA, Waxman SG (2006). A single sodium channel mutation produces hyper- or hypoexcitability in different types of neurons. Proc Natl Acad Sci USA.

